# Heterogeneity in Self-Reported Management of Blunt Chest Trauma: An International Cross-Sectional Survey

**DOI:** 10.3390/jcm15197668

**Published:** 2026-10-03

**Authors:** Matteo Cimino, Salvatore Notaro, Antonio M. Esquinas, Marina Attanasi, Michela Leone, Yusuf Kahya, Giulia Michela Pellegrino, Matjaž Groznik, Adel Mohamad Alansary, Abdullah Ali Al-Bishari, Jose Luis Sandoval Gutierrez, Mostafa Elshazly, Sanjay Singhal, Mohsen Sadeghi, Federico Raimondi, Emanuele Voulaz, Claudia Crimi, Marianna Porzio, Michele Umbrello, Daniele Russo, Hayato Kurihara, Giuseppe Fiorentino, Bushra Mina, Fabiano Di Marco, Giuseppe Francesco Sferrazza Papa, on behalf of the Chest Trauma Study Group

**Affiliations:** 1 Emergency Surgery Unit, Fondazione IRCCS Ca’ Granda Ospedale Maggiore Policlinico, 20141 Milan, Italy; matteo.cimino@policlinico.mi.it (M.C.); hayato.kurihara@policlinico.mi.it (H.K.); 2 Intensive Care Unit and ECMO Center, AORN Ospedali dei Colli, Monaldi Hospital, 80131 Naples, Italy; 3 International School of Doctorate, EIDUM, University of Murcia, 30100 Murcia, Spain; 4 Intensive Care Unit, Hospital Morales Meseguer NIV-ICU Group, Biomedical Research Institute Pascual Parrilla–IMIB, 30008 Murcia, Spain; antmesquinas@gmail.com; 5 Pediatric Allergy and Pulmonology Unit, Department of Pediatrics, University of Chieti-Pescara, 66100 Chieti, Italy; marina.attanasi@unich.it; 6 Intensive Care Unit, Fondazione IRCCS Ca’ Granda Ospedale Maggiore Policlinico, 20141 Milan, Italy; michela.leone@policlinico.mi.it; 7 Department of Thoracic Surgery, Ibni Sina Hospital, Ankara University School of Medicine, Ankara 06230, Turkey; dr.yusufkahya@hotmail.com (Y.K.); francesco.sferrazza@gmail.com (G.F.S.P.); 8 Department of Neurorehabilitation Sciences, Casa di Cura Igea, 20144 Milan, Italy; g.pellegrino@casadicuraigea.it; 9 Department of Traumatology, University Clinical Center Ljubljana, 1000 Ljubljana, Slovenia; matjazgroznik@gmail.com; 10 Department of Anesthesiology and Critical Care, Faculty of Medicine, Ain Shams University, Cairo 11566, Egypt; adelalansary@med.asu.edu.eg; 11 Respiratory Therapy Department, 22 May Hospital, Sana’a, Yemen; drabduallahalialbeshari@gmail.com; 12 Mexican Society of Pulmonology and Thoracic Surgery, Mexico City 03810, Mexico; sandovalgutierrez@gmail.com; 13 Kasr Al Ainy School of Medicine, Cairo University, Cairo 11562, Egypt; elshazly66@hotmail.com; 14 Dr. Ram Manohar Lohia Institute of Medical Sciences, Lucknow 226010, India; drsanjaysinghal79@yahoo.co.in; 15 Inflammatory Lung Disease Research Center, Department of Internal Medicine, Razi Hospital, School of Medicine, Guilan University of Medical Sciences, Rasht 41448-95655, Iran; doctormsadeghi@gmail.com; 16 Chronic Respiratory Diseases Research Center, National Research Institute of Tuberculosis and Lung Diseases, Shahid Beheshti University of Medical Sciences, Tehran 19569-44413, Iran; 17 Respiratory Medicine Unit, ASST Papa Giovanni XXIII, 24127 Bergamo, Italy; fraimondi@asst-pg23.it; 18 Division of Thoracic Surgery, IRCCS Humanitas Research Hospital, Rozzano, 20089 Milan, Italy; emanuele.voulaz@humanitas.it; 19 Department of Biomedical Sciences, Humanitas University, Pieve Emanuele, 20090 Milan, Italy; 20 Department of Clinical and Experimental Medicine, University of Catania, 95123 Catania, Italy; claudia.crimi@unict.it; 21 Respiratory Medicine Unit, AOU Policlinico “G. Rodolico–San Marco”, 95124 Catania, Italy; 22 Fondazione IRCCS Ca’ Granda Ospedale Maggiore Policlinico, 20141 Milan, Italy; marianna.porzio@policlinico.mi.it; 23 SC Rianimazione e Anestesia, ASST Ovest Milanese, Ospedale Civile di Legnano, 20025 Legnano, MI, Italy; michele.umbrello@ao-sanpaolo.it; 24 Department of Pediatrics, University of Chieti-Pescara, 66100 Chieti, Italy; danielerusso1607@gmail.com; 25 Pediatric Allergy and Respiratory Unit, Department of Pediatrics, University of Chieti-Pescara, 66100 Chieti, Italy; 26 Respiratory Pathophysiology Unit, AORN Ospedali dei Colli, Monaldi Hospital, 80131 Naples, Italy; giuseppe.fiorentino@ospedalideicolli.it; 27 Department of Translational Medical Sciences, Federico II University, 80131 Naples, Italy; 28 Division of Pulmonary, Critical Care and Sleep Medicine, Northwell Health, New York, NY 11040, USA; bminamd@gmail.com; 29 Zucker School of Medicine at Hofstra/Northwell, Hempstead, NY 11549, USA; 30 Department of Health Sciences, University of Milan, 20122 Milan, Italy; fabiano.dimarco@unimi.it; 31 Respiratory Unit and Department of Organ Failure and Transplantation, ASST Papa Giovanni XXIII, 24127 Bergamo, Italy

**Keywords:** blunt chest trauma, chest trauma, non-invasive ventilation, rib fractures, rib-fracture management, survey

## Abstract

**Background**: Blunt chest trauma is common, but reported management practices may differ across clinical settings. This international cross-sectional survey evaluated self-reported practice patterns in the diagnosis and management of blunt chest trauma. **Methods**: We distributed a self-administered web-based questionnaire to healthcare professionals involved in chest trauma care between November 2024 and August 2025. Responses were analyzed descriptively, and exploratory comparisons were performed between Italian respondents and respondents from other participating countries. **Results**: A total of 509 responses were included, comprising 225 Italian and 284 international respondents. Within the participating sample, differences between Italian respondents and respondents from other participating countries were observed in institutional protocol availability, diagnostic imaging choices, pain management organization, adjunctive therapies, and factors guiding rib-fracture management. Greater consistency was observed in chest tube placement and escalation of respiratory support. **Conclusions**: Substantial heterogeneity in self-reported blunt chest trauma management was observed among participating clinicians, particularly regarding institutional protocols, diagnostic strategies, pain management, adjunctive therapies, and rib-fracture management. Greater consistency was observed in core interventions such as chest tube placement and escalation of respiratory support. Given the voluntary sampling strategy and unequal geographic representation, these findings should be interpreted as patterns within the participating sample rather than as representative estimates of national or global practice.

## 1. Introduction

Blunt chest, or thoracic, trauma accounts for approximately 10–15% of trauma presentations to emergency departments [1,2,3]. In a large cohort of trauma patients with thoracic injuries, blunt trauma accounted for 84% of cases [4]. The management of these injuries is of broad multidisciplinary interest, involving emergency physicians and surgeons, pulmonologists, thoracic surgeons, anesthesiologists, and intensive care specialists caring for both adults and children. While overall mortality can reach 25%, it is generally lower in isolated cases, where adverse outcomes are often driven by associated injuries—such as traumatic brain or abdominal injuries—and secondary complications, including Acute Respiratory Distress Syndrome (ARDS) and pneumonia [1,2,3,4]. Historically, management focused on analgesia and respiratory physiotherapy as the primary approaches to reduce complications. However, recent advances in positive-pressure ventilation and the introduction of surgical stabilization of rib fractures (SSRF) have shifted the therapeutic landscape.

Yet, treatment strategies remain largely non-standardized. Several risk scores have been proposed and validated, which implies that similar chest blunt trauma is classified differently. Patients with low and moderate risk are discharged without outpatient follow-up, and a proportion of them will re-attend the Emergency Department (ED) with secondary complications such as chronic pain or pneumonia. Furthermore, the role of SSRF is still a subject of debate; while traditionally reserved for flail chest, emerging evidence shows possible benefits for patients with multiple rib fractures without flail chest [5,6]. Pulmonary physiotherapy is widely advocated as a useful tool to improve long-term outcomes [7,8], but no uniform protocols have been disseminated. In recent years, patient-reported outcome measures and quality-of-life questionnaires [9,10] have been increasingly utilized to understand better long-term recovery and the impact of treatment on patients’ daily lives [11].

Recent international guidance has sought to standardize key aspects of thoracic trauma management. The WSES-AAST thoracic trauma guidelines provide comprehensive recommendations for the management of thoracic injuries, while the WSES/CWIS position paper provides updated consensus statements on the indications, timing, imaging assessment, and operative management of surgical stabilization of rib fractures. Nevertheless, the implementation of these recommendations across different specialties and clinical settings remains incompletely characterized [12,13].

This study aimed to describe heterogeneity in self-reported approaches to blunt chest trauma management among clinicians participating in an international survey. We also explored differences between Italian respondents and respondents from other participating countries to identify areas of practice variation that may warrant further investigation and future standardization efforts.

## 2. Materials and Methods

We conducted a cross-sectional, self-administered, web-based survey of health professionals involved in the management of patients with blunt chest trauma between November 2024 and August 2025.

### 2.1. Questionnaire Development and Testing

The questionnaire was developed by a multidisciplinary scientific committee comprising an emergency surgeon (MC), a pulmonologist (GFSP), anesthesiologists (SN and ML), an emergency physician (MP), an intensivist (MU), a thoracic surgeon (EV), and a pediatrician (MA). Survey domains and individual items were generated through a review of the relevant literature and focus-group discussions within the scientific committee. Items were subsequently reviewed and reduced to ensure alignment with the study objectives while minimizing respondent burden.

The questionnaire was pilot-tested by 10 physicians, including surgeons, respiratory physicians, intensivists, and residents who were not involved in its development. Based on their feedback, minor revisions were made before final administration. The final questionnaire is provided in Supplementary Material S1 and comprised 32 study-related questions organized into six key domains:

1. Preferred initial imaging modality (chest X-ray, chest computed tomography, or thoracic ultrasound) in hemodynamically stable and unstable patients.
2. Management of pneumothorax, including observation, needle aspiration, and chest tube placement.
3. Use of non-invasive ventilation (NIV) and invasive mechanical ventilation (IMV).
4. Availability of Acute Pain Service (APS) and preferred analgesic strategies for rib fractures.
5. Criteria for Intensive Care Unit (ICU) admission and use of chest trauma severity scores.
6. Medical and surgical management of blunt chest trauma.

Response options included nominal, ordinal, and interval responses.

Sampling Frame and Questionnaire Administration

The survey was administered in English using the SurveyMonkey platform. Some questions were mandatory, whereas others were optional. Branching logic was applied so that follow-up questions were displayed only when relevant on the basis of previous responses; consequently, item-specific denominators varied across questions. SurveyMonkey settings were enabled to limit repeated submissions. No translated versions of the questionnaire were used. The questions were developed according to CROSS and CHERRIES recommendations.

The target population included healthcare professionals involved in the management of blunt chest trauma, including anesthesiologists, intensivists, trauma surgeons, pulmonologists, emergency physicians, and pediatricians. Potential respondents were recruited through international respiratory and critical care societies, including the Italian Respiratory Society, the International NIV School in Spain, the European Respiratory Society, and the Turkish Thoracic Society, as well as through several international collaborators with expertise in respiratory medicine, intensive care, emergency medicine, anesthesiology, and surgery from multiple countries. These collaborators facilitated survey dissemination through their local and international professional networks and helped distribute the survey. These organizations did not participate in questionnaire development, data analysis, interpretation of the findings, or manuscript preparation.

The survey was administered electronically using the SurveyMonkey platform and distributed through professional societies and investigator networks. An initial invitation was followed by a reminder one month later and a final reminder three months after the initial invitation. Participation was voluntary and anonymous. The survey remained open until 31 August 2025. The complete questionnaire is provided in Supplementary Material S1. Italy was analyzed separately because it represented the largest single-country subgroup in the survey (n = 225). Comparisons between Italian respondents and respondents from other participating countries were considered exploratory within-sample comparisons and were not intended to provide representative estimates of differences between national healthcare systems.

Because voluntary snowball sampling was used without a prespecified sampling frame, we were unable to assess the response rate or non-response bias. As participation was voluntary and recruitment occurred through professional societies and investigator networks, selection bias cannot be excluded.

### 2.2. Statistical Analysis

Data were collected, compiled, and analyzed using IBM SPSS Statistics, version 31.0 (IBM Corp., Armonk, NY, USA). Descriptive statistics, including frequencies and proportions, were used to summarize survey responses and respondents’ characteristics. Categorical variables are presented as frequencies and percentages. Comparisons between Italian and international respondents were conducted using the chi-square test or Fisher’s exact test, as appropriate. Because this was an exploratory descriptive survey based on a non-probability snowball sample, with no prespecified primary outcome and substantial heterogeneity between the comparison groups, we did not calculate effect sizes or 95% confidence intervals, as these estimates could misleadingly imply a degree of population-level precision and generalizability that the sampling design does not support; accordingly, all between-group comparisons are explicitly presented as exploratory and interpreted with caution.

### 2.3. Ethics Statement

Participation in the survey was voluntary and anonymous.

Ethics committee approval was not sought because this study consisted of a voluntary, anonymous survey of healthcare professionals, involved no patients or clinical interventions, and collected no patient-level, identifiable, or sensitive personal data.

Responses were analyzed only in aggregate form.

## 3. Results

### 3.1. Respondent Characteristics

The questionnaire was distributed from 30 November 2024 to 31 August 2025. A total of 509 responses were included in the analysis. Not all respondents completed the whole questionnaire, so the denominators may vary according to the rate of response.

**Figure 1:**
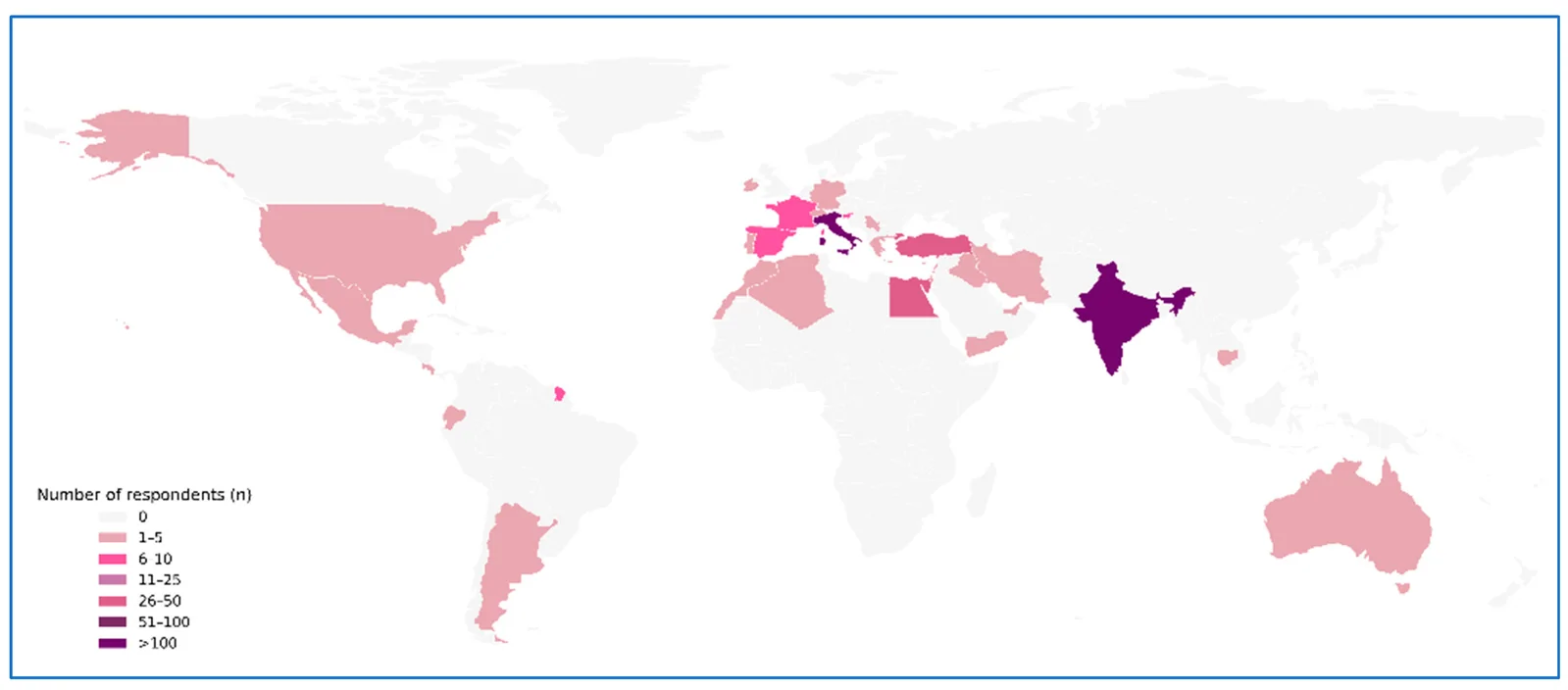
Geographical distribution of survey respondents according to country of origin. Colors indicate the number of respondents from each country.

Country of practice was reported by 496 of the 509 respondents. Respondents with available country information represented 29 countries. Italy represented the largest single-country group (n = 225), followed by India (n = 142), Turkey (n = 42), Egypt (n = 39), France (n = 9), Spain (n = 9), Slovenia (n = 7), Yemen (n = 2), and 21 additional countries represented by one respondent each. Country information was unavailable for 13 internationals respondents. The geographical distribution is shown in Figure 1.

The age distribution differed modestly between the two groups, with most participants in both cohorts falling within the 31–40 years of age category (Table 1).

Male respondents represented the majority in both groups.

### 3.2. Professional Background and Training

The two cohorts differed in professional training level. Regarding professional specialty, the two cohorts showed different distributions. Anesthesiology was more frequently represented among Italian respondents, whereas intensive care and pulmonology were more frequently represented among international respondents.

### 3.3. Hospital Characteristics

Institution type differed between the two groups. International respondents more frequently reported working in third-level hospitals, whereas Italian respondents were more commonly affiliated with second-level hospitals.

### 3.4. Availability of Organizational Procedures

A marked difference was observed in the availability of organizational procedures for chest trauma management. An internal NIV protocol was reported by 58.1% of international respondents compared with 29.0% of Italian respondents.

### 3.5. Self-Reported Expertise

Participants rated their level of expertise in managing chest wall trauma. Italian respondents more frequently classified themselves as non-experts, while a higher proportion of international respondents reported being experienced or expert clinicians in this field.

### 3.6. Diagnostic Approach

**Figure 2:**
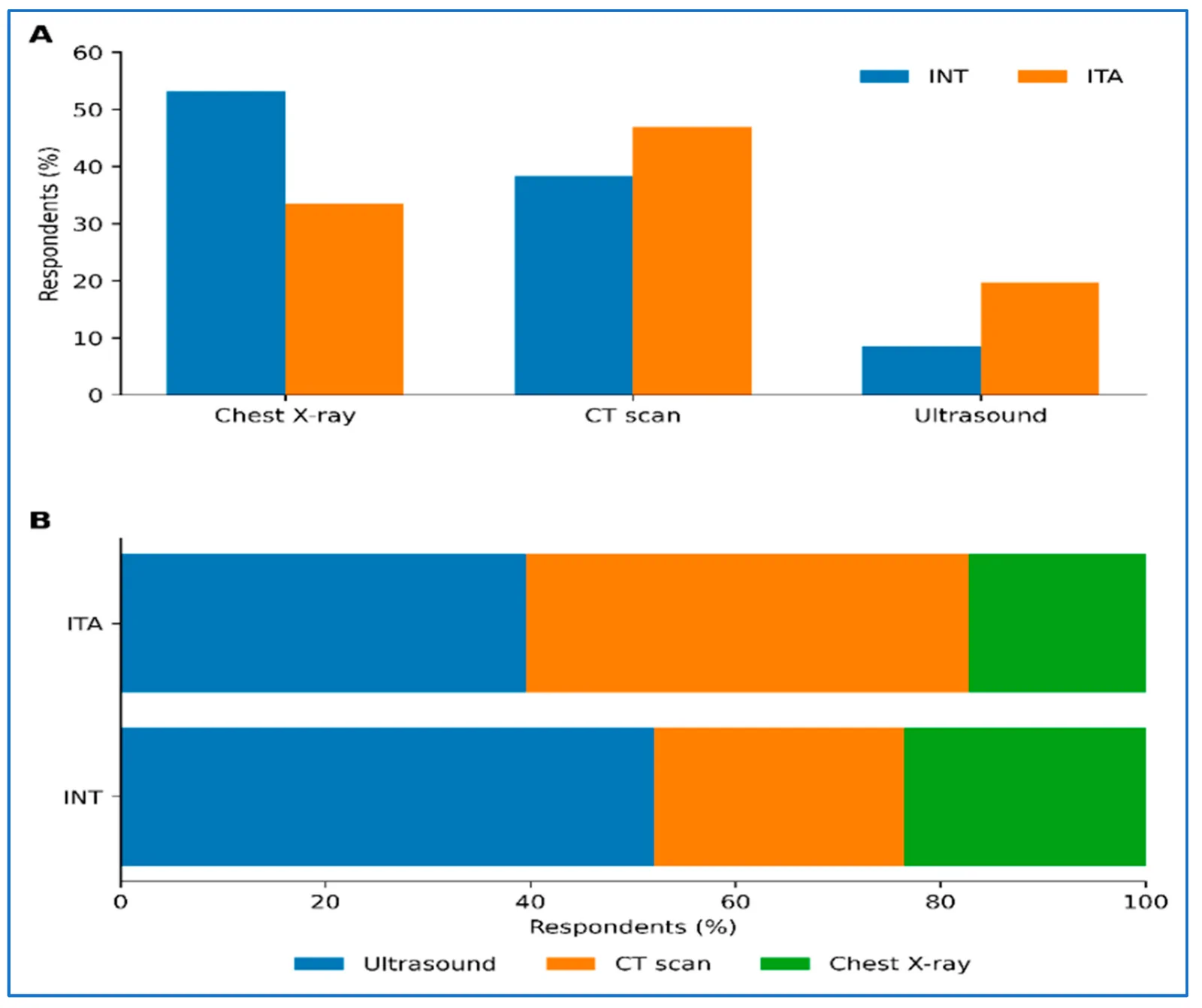
Preferred first-line imaging modality according to hemodynamic status. (**A**) Preferred imaging modality in hemodynamically stable patients. (**B**) Preferred imaging modality in hemodynamically unstable patients. ITA, Italian respondents; INT, international respondents.

Differences were observed in the preferred initial imaging modality according to hemodynamic status (Table 2). In hemodynamically stable patients, chest X-ray was more frequently selected by international than Italian respondents (53.2% vs. 33.5%), whereas CT scanning was more frequently selected by Italian respondents (46.9% vs. 38.4%). In hemodynamically unstable patients, ultrasound was more frequently selected by international respondents (52.1%), whereas Italian respondents showed a more balanced distribution between CT scanning (43.1%) and ultrasound (39.6%) (Figure 2).

The most commonly reported severity scores were the Chest Trauma Score (40.3% of international respondents vs. 47.7% of Italian respondents) and the Thoracic Trauma Severity Score (41.1% vs. 28.7%, respectively).

### 3.7. Blunt Chest Trauma Management

**Figure 3:**
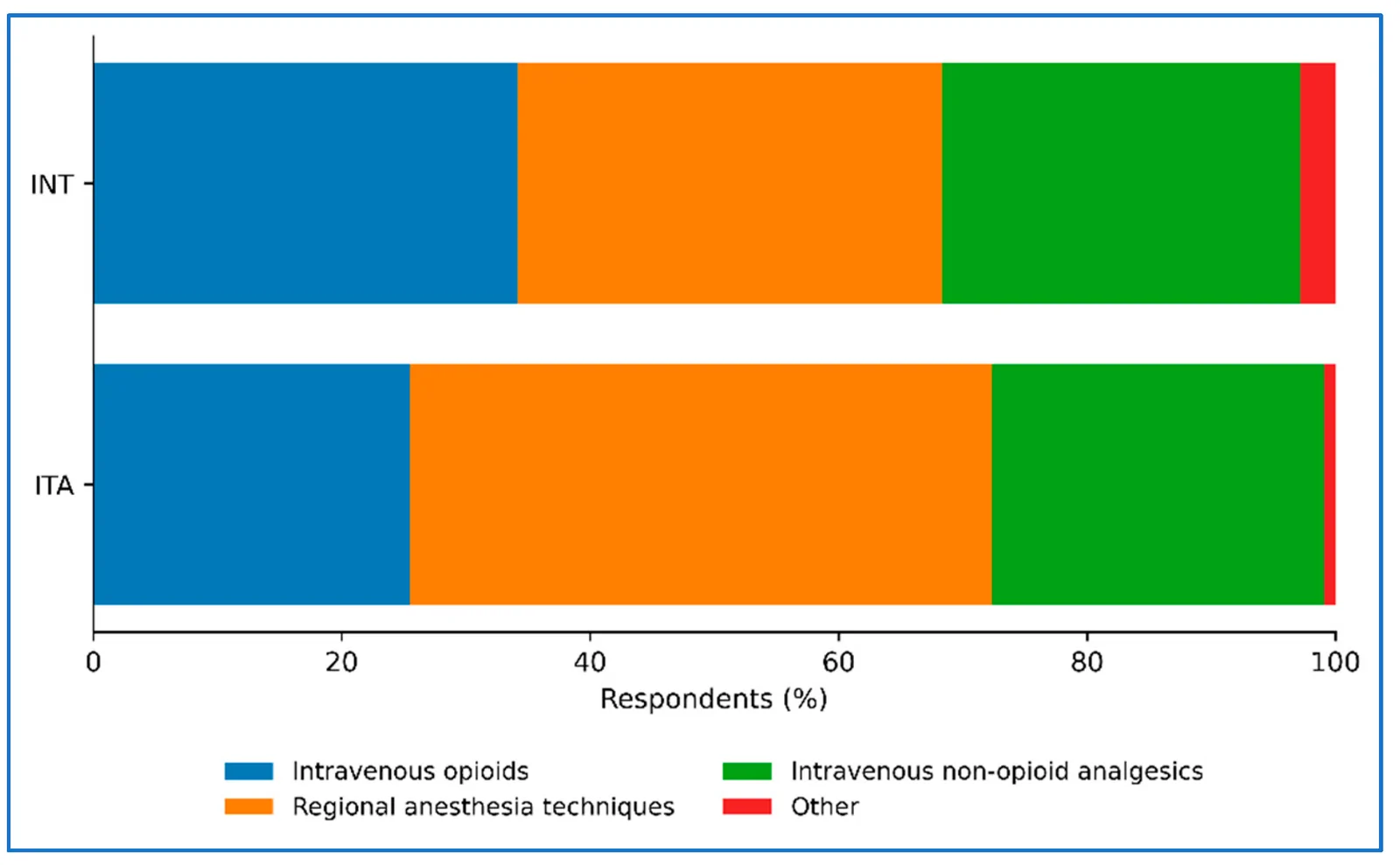
Analgesic strategies routinely adopted for the management of blunt chest trauma among Italian (ITA) and international (INT) respondents.

Pain control strategies varied between groups, particularly regarding the involvement of Acute Pain Service (APS). A dedicated APS was available in 59.9% of international centers compared with 75.1% of Italian centers. Regional anesthesia techniques were more frequently selected by Italian respondents than by international respondents (46.9% vs. 34.2%). Among international respondents, regional anesthesia techniques and intravenous opioids were equally selected (34.2% each), whereas intravenous opioids were selected by 25.5% of Italian respondents (Figure 3).

Chest tube placement was similarly reported across both cohorts. In hemodynamically stable patients with pneumothorax, chest tube insertion was reported by 65.7% of international and 66.4% of Italian respondents. In hemodynamically unstable patients, the corresponding proportions were 70.7% and 71.0%, respectively (Table 2).

Regarding initial respiratory support, NIV was the most frequently selected modality among international respondents (47.9%), followed by HFNO (27.5%). Among Italian respondents, HFNO (39.9%) and NIV (37.7%) were the most frequently selected modalities (Table 3).

**Figure 4:**
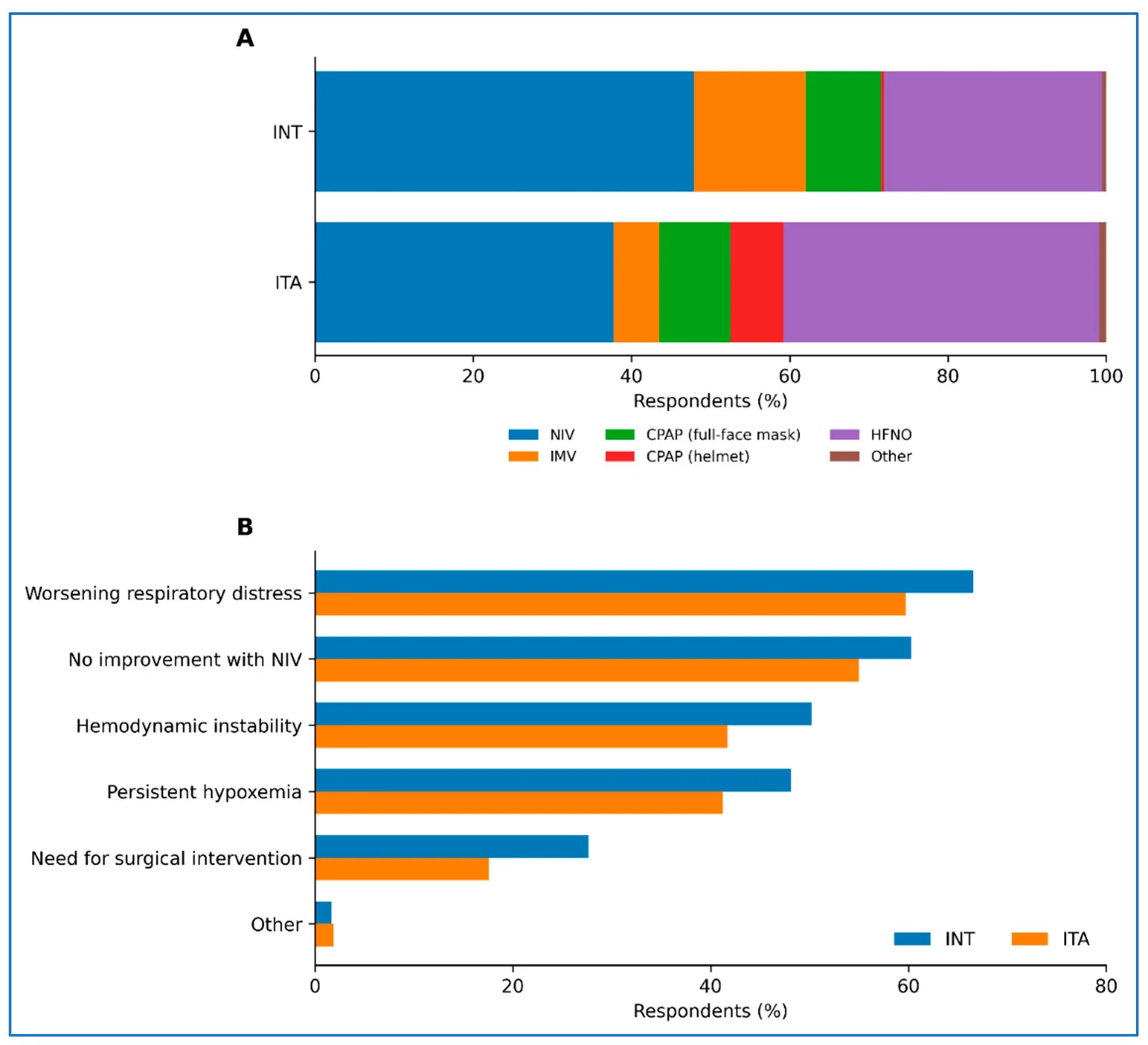
Respiratory support strategies in blunt chest trauma. (**A**) Preferred initial respiratory support modality among Italian (ITA) and international (INT) respondents. (**B**) Reported indications for transition from non-invasive ventilation (NIV) to invasive mechanical ventilation (IMV). CPAP, continuous positive airway pressure; HFNO, high-flow nasal oxygen.

IMV was less frequently selected in both groups (14.1% of international and 5.8% of Italian respondents). Worsening respiratory distress was the most frequently reported indication for transition from NIV to IMV (66.5% of international and 59.7% of Italian respondents), followed by failure to improve with NIV (60.3% and 55.0%, respectively) (Figure 4).

Pneumothorax was not considered an absolute contraindication to NIV by 62.2% of international respondents and 75.9% of Italian respondents.

### 3.8. Adjunctive Therapies

Among respondents to the item on adjunctive therapies in patients receiving NIV (229 international and 208 Italian respondents), physiotherapy (47.2% vs. 26.4%) and bronchodilators (45.9% vs. 14.4%) were more frequently reported by international respondents, whereas diuretics (9.2% vs. 32.2%) were more frequently reported by Italian respondents.

### 3.9. Decision-Making Factors for Rib Fracture Surgical Stabilization

**Figure 5:**
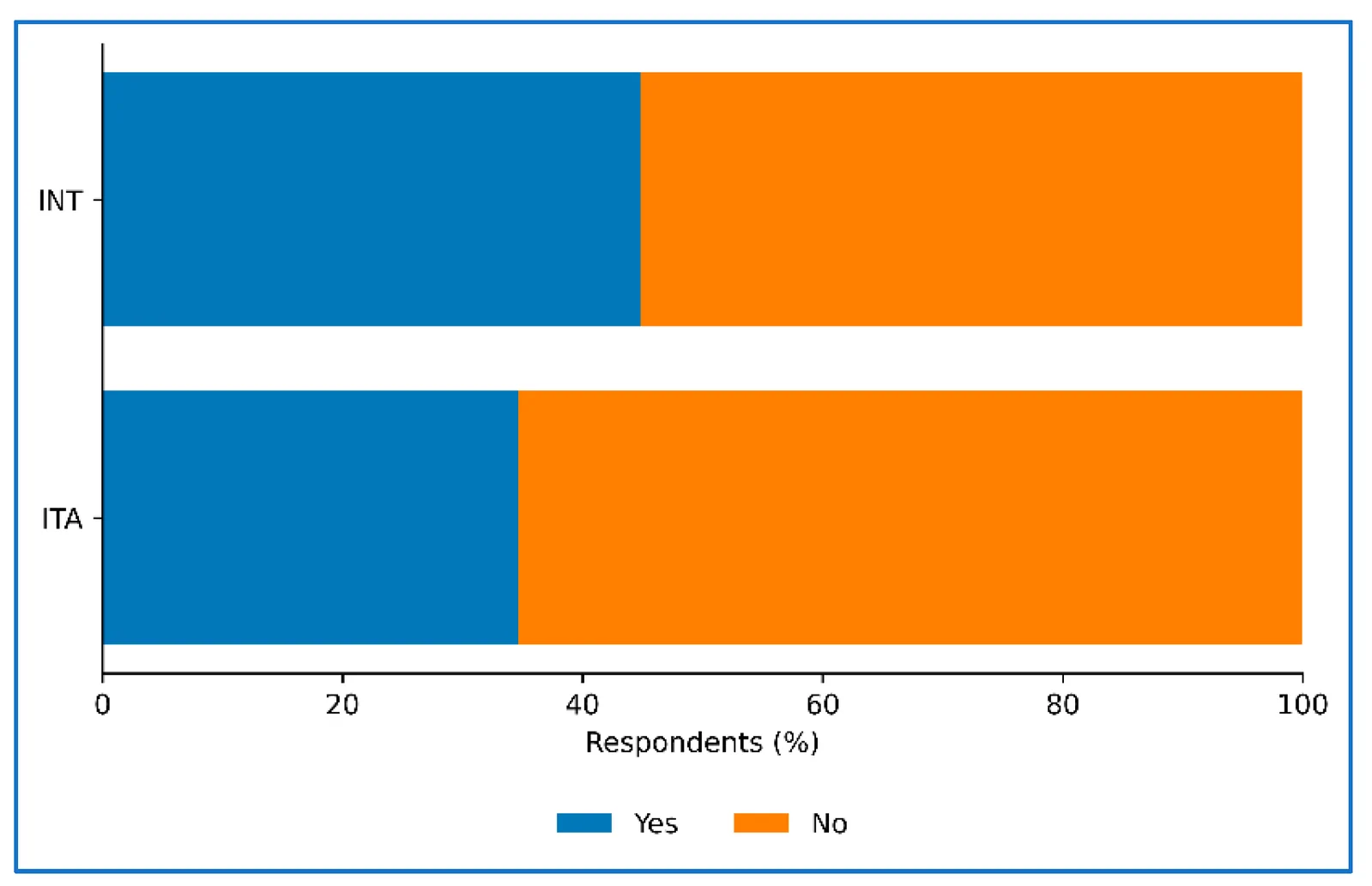
Use of an institutional diagnostic and management flowchart for rib fractures among Italian (ITA) and international (INT) respondents.

The use of an institutional diagnostic and management flowchart for rib fractures was reported by 44.8% of international respondents and 31.6% of Italian respondents (Figure 5).

When asked to identify the main factor guiding rib-fracture treatment, 166 of 222 international respondents (74.8%) selected flail chest, whereas 142 of 199 Italian respondents (71.4%) selected patient age (Table 3). These responses describe the factors respondents considered most relevant to rib-fracture management; the questionnaire item did not distinguish between operative and nonoperative treatment.

**Figure 6:**
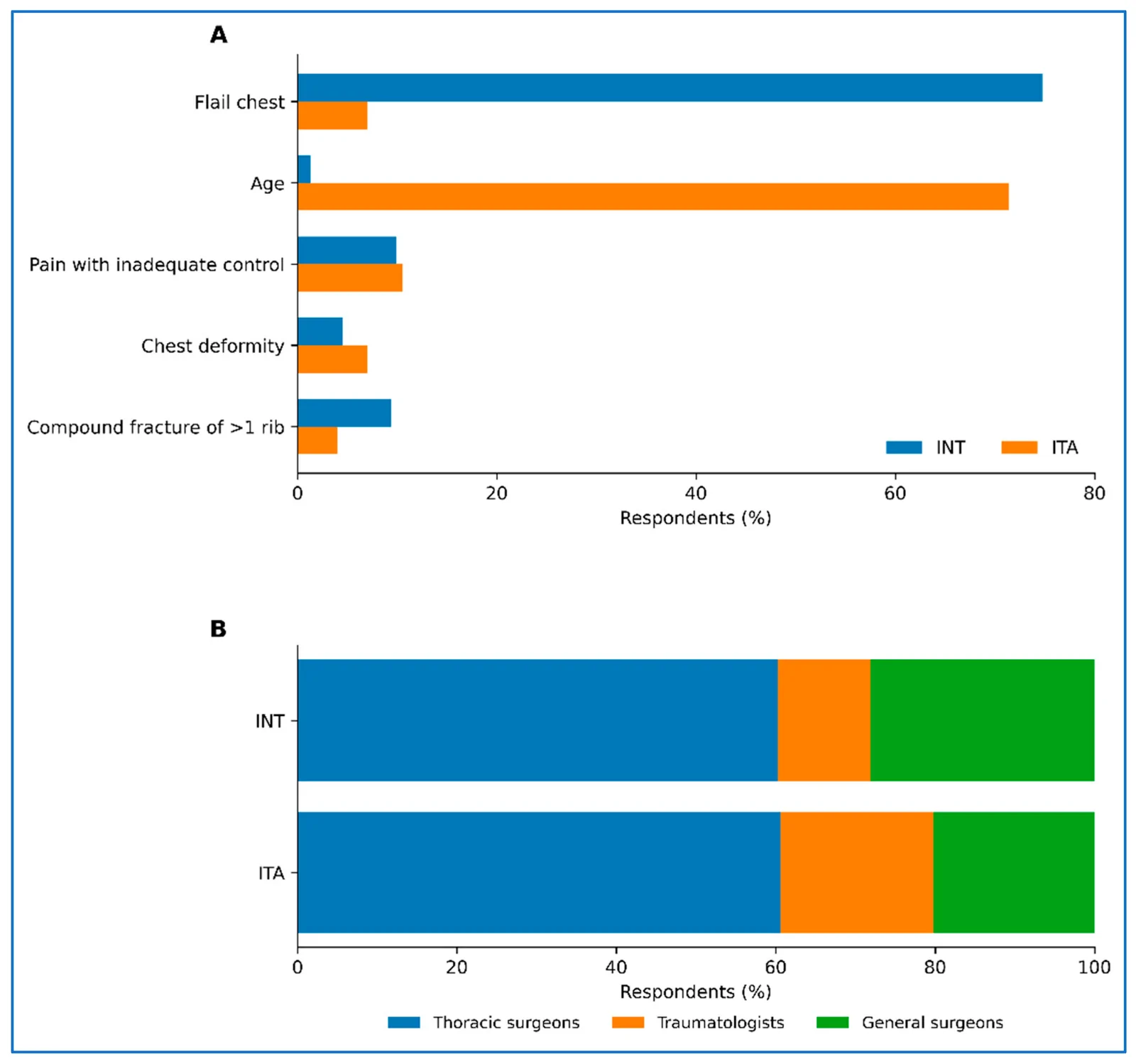
Rib fracture management according to respondent group. (**A**) Factors guiding treatment decisions for rib fractures among Italian (ITA) and international (INT) respondents. (**B**) Specialists primarily responsible for the management of rib fractures among Italian and international respondents.

Thoracic surgeons were the specialists most frequently reported as responsible for rib fracture management in both groups (60.3% of international and 60.6% of Italian respondents). General surgeons were more frequently reported by international respondents (28.1% vs. 20.2%), whereas traumatologists were more frequently reported by Italian respondents (19.2% vs. 11.6%) (Figure 6).

## 4. Discussion

This international survey identified considerable heterogeneity in self-reported approaches to blunt chest trauma management among participating clinicians. Differences were observed between Italian respondents and respondents from other participating countries across several domains, including protocol availability, diagnostic strategies, adjunctive therapies, and management decisions. Given the voluntary sampling strategy, unequal country representation, and marked differences in respondent and institutional characteristics, these findings should be interpreted as within-sample patterns rather than as representative differences between national healthcare systems. One of the most relevant findings is the limited availability of organizational procedures in Italy compared to the international cohort. Less than one-third of Italian respondents reported having an internal NIV protocol, compared with more than half of international clinicians. These differences may be influenced by the markedly different professional and institutional characteristics of the participating groups and should therefore not be interpreted as direct evidence of differences between healthcare systems. Differences in professional background and training may also contribute to the observed variability. The distribution of medical specialties differed between the Italian and international cohorts, which may influence approaches to chest trauma management and treatment decisions.

Another main finding relates to diagnostic strategies, with Italian clinicians that seem to prefer early computed tomography than international respondents, who more frequently reported chest X-ray as the initial imaging modality. This difference may reflect variations in resource availability, clinical pathways, expertise, or risk tolerance, and is consistent with continuing trends toward increased use of advanced imaging in trauma care [14].

The present survey shows considerable variability in pain control methods, particularly in the use of Acute Pain Service (APS) and the choice between regional and systemic analgesia. Interestingly, Italian respondents reported greater availability of APS (75.1% vs. 59.9%) and greater use of regional analgesic techniques. In contrast, international clinicians showed a more balanced distribution between regional approaches and intravenous opioids.

These outcomes are consistent with the current literature, which increasingly supports the use of multimodal and opioid-sparing analgesia in patients with chest trauma. Recent reviews emphasize that combining systemic analgesics with regional techniques is the most effective strategy for pain control and prevention of pulmonary complications [15]. Adequate pain control is essential to prevent hypoventilation, atelectasis, and pneumonia, all of which are strongly associated with poor outcomes in patients with rib fractures.

The higher use of regional analgesia observed in the Italian cohort is consistent with growing evidence favoring these techniques. Thoracic epidural analgesia has traditionally been regarded as a reference technique, providing effective pain control and improved respiratory mechanics [16]. However, more recent data have questioned its universal superiority, highlighting limitations such as technical complexity, contraindications, and potential complications [17]. In parallel, newer regional techniques—such as paravertebral, erector spinae plane (ESP), and serratus anterior plane (SAP) blocks—have gained increasing popularity due to their favorable safety profile and ease of use, additionally supporting the trend toward regional analgesia observed in our survey [18].

These techniques may also help prevent chronic pain [19,20].

Conversely, the continued use of intravenous opioids, particularly among international respondents, reflects their established role in acute pain control. Opioids remain one of the most studied and widely used analgesic classes in rib fracture management; however, their use is increasingly tempered by concerns regarding respiratory depression, delirium, and other adverse effects, especially in elderly patients [15]. This has led to an increasing emphasis on opioid-sparing strategies, which may explain the relatively lower reliance on systemic opioids observed in the Italian cohort.

Importantly, the variability observed in our study is consistent with the broader literature, which highlights a lack of high-quality evidence and uniform protocols for pain management in chest trauma. Systematic reviews have demonstrated heterogeneity in analgesic approaches—including epidural, intravenous, and regional techniques—with inconclusive results regarding superiority in terms of mortality and major clinical outcomes [21].

As a result, current practice remains largely influenced by local expertise, resource availability, and institutional organization, rather than universally accepted guidelines.

Overall, our findings support the concept that while multimodal analgesia is widely recognized as a key component of chest trauma management, its implementation differs substantially across healthcare settings.

The higher integration of APS and greater use of regional techniques observed in Italy may reflect differences in the organization and delivery of pain management services. In contrast, international practice appears more heterogeneous, balancing regional and systemic strategies.

Another relevant finding of the present study is the frequent use of non-invasive ventilation (NIV), with relatively limited use of invasive mechanical ventilation (IMV). This pattern is consistent with previous evidence supporting the use of NIV in appropriately selected patients with chest trauma-related respiratory failure. A systematic review by Chiumello et al. demonstrated that NIV is associated with a significant reduction in mortality and intubation rates compared with standard therapy in patients with chest trauma-related respiratory failure [22]. Similarly, more recent meta-analyses suggest that NIV may reduce mortality and complications compared with IMV [23].

Importantly, the frequently reported use of both NIV and chest tube placement in our survey reflects a clinically relevant aspect of chest trauma management. While pneumothorax has traditionally been considered a contraindication to NIV, recent evidence suggests a more nuanced approach.

A contemporary multicenter study demonstrated that NIV can be safely applied in chest trauma patients, with a low incidence of pneumothorax progression, particularly when appropriate pleural drainage is performed. In that study, worsening pneumothorax occurred in only 1.3% of patients without prior pneumothorax. It remained relatively low even among those with pre-existing pneumothorax, especially when a chest tube was in place [24].

This is consistent with our data, where chest tube utilization was nearly identical between cohorts. These findings support the concept that pneumothorax should not necessarily be considered an absolute contraindication to NIV, but rather a condition requiring appropriate assessment and management before or during ventilatory support.

Furthermore, earlier systematic reviews have shown that NIV is associated with lower complication rates, shorter ICU stays, and reduced mortality compared to IMV, although the quality of evidence remains heterogeneous. This uncertainty in the literature likely contributes to the variability observed in our survey, particularly in the adoption of ventilatory strategies across different healthcare systems [25].

Variability was also observed in the use of adjunctive therapies, with international respondents more frequently reporting respiratory-oriented interventions, such as bronchodilators and physiotherapy. In contrast, Italian clinicians reported a higher use of diuretics.

This finding illustrates the broader uncertainty in the literature regarding adjunctive treatment strategies in chest trauma. Current evidence supports pulmonary physiotherapy and early mobilization as part of multimodal care, with studies suggesting reduced pulmonary complications such as atelectasis and pneumonia. These interventions are commonly included in rib fracture management guidelines, although the quality of the available evidence remains limited [26].

Similarly, bronchodilators are frequently used in clinical practice but are supported by limited direct evidence in trauma populations, with most data extrapolated from other respiratory conditions. Their use may reflect an attempt to optimize airway function and secretion clearance rather than a guideline-driven indication; however, evidence supporting their routine use in this setting remains scarce [27].

In contrast, the higher use of diuretics observed among Italian respondents is less clearly supported by current evidence. While diuretics may be considered in selected cases of pulmonary contusion associated with fluid overload, they are not routinely recommended in trauma guidelines. Contemporary trauma care strategies instead emphasize careful fluid management and avoidance of both under- and over-resuscitation, rather than pharmacological fluid removal [28].

The different factors prioritized by the two respondent groups should be interpreted within the scope of the questionnaire item. Respondents were asked about a factor guiding rib-fracture treatment, but were not asked whether they would perform surgical stabilization of rib fractures (SSRF). Therefore, these findings cannot establish respondents’ indications for SSRF or how frequently they use it. Differences in specialty and institutional characteristics between the groups, as well as differences in how the question was interpreted, may have influenced the responses. A survey that explicitly separates operative from nonoperative decisions would be needed to investigate practice patterns concerning SSRF.

These findings are consistent with the current literature. Evidence supports the use of SSRF in selected patients with flail chest, with randomized controlled trials and meta-analyses reporting reductions in mechanical ventilation duration, ICU length of stay, and pneumonia incidence [6].

In contrast, the role of SSRF in patients with non-flail rib fractures remains less clearly defined. Although recent studies indicate possible benefits in selected patients with severe non-flail rib fracture patterns [5], the available evidence remains heterogeneous, and universally accepted indications have not yet been established. This uncertainty is reflected in the variability observed in our survey, particularly among Italian respondents, where decision-making appears to be more strongly influenced by patient characteristics rather than injury morphology alone.

The greater emphasis on patient-related factors, such as age, may reflect a more conservative or individualized approach, perhaps influenced by concerns regarding surgical risk, comorbidities, and resource availability. Conversely, the injury-focused approach observed among international respondents may be associated with greater exposure to dedicated trauma systems and higher familiarity with SSRF techniques.

The present study has several additional limitations. First, the survey relied on self-reported practices rather than observed clinical behavior; therefore, responses may reflect perceived or ideal management strategies rather than actual bedside practice. Second, participation was voluntary, introducing the possibility of selection bias, as clinicians with a particular interest or expertise in chest trauma may have been more likely to respond. In addition, because a prespecified sampling frame was not available, the response rate and potential non-response bias could not be assessed. Third, distributing the survey through scientific societies and professional networks may have resulted in unequal geographical representation, with some countries and healthcare systems overrepresented while others were underrepresented. An additional limitation concerns the comparison between Italian respondents and the pooled group of respondents from other participating countries. The latter group was geographically heterogeneous, and the two comparison groups differed substantially in specialty, professional experience, self-reported expertise, hospital characteristics, and working setting. Consequently, crude between-group differences may partly reflect differences in respondent composition and residual confounding rather than geographic or healthcare-system effects. In addition, potential clustering of respondents within countries and institutions should be considered when interpreting country-level comparisons. The rib-fracture item did not distinguish operative from nonoperative management and therefore cannot establish respondents’ indications for surgical stabilization of rib fractures.

Fourth, although the questionnaire was piloted before dissemination, some questions may have been interpreted differently across specialties, languages, and healthcare environments, potentially affecting response consistency. Fifth, the survey did not collect detailed information regarding local resource availability, trauma volume, access to thoracic surgery, or institutional case-mix, factors that may substantially influence clinical decision-making and partially explain the observed variability. Finally, because the study was cross-sectional, it provides a snapshot of contemporary practice but cannot assess temporal changes in management strategies or determine whether any particular approach is associated with superior clinical outcomes.

In spite of these limitations, our study shows greater consistency in core interventions, particularly chest tube placement and escalation of respiratory support.

This finding is consistent with the broader trauma literature, in which fundamental principles such as management of pneumothorax and respiratory failure are widely standardized even when detailed local protocols are absent.

Taken together, our findings both confirm and extend existing evidence. While previous studies have examined individual aspects of chest trauma management, our survey provides insight into real-world clinical decision-making, showing how clinicians integrate NIV, pain management, chest tube placement, nonoperative management optimization, and SSRF within daily practice. The observed consistency in these core interventions, despite variability in other domains, suggests that clinicians share a common physiological framework even without unified guidelines.

## 5. Conclusions

This international survey identified substantial heterogeneity in self-reported blunt chest trauma management among participating clinicians, particularly regarding institutional protocols, diagnostic strategies, pain management, adjunctive therapies, and rib-fracture management. Greater consistency was observed in core interventions, including chest tube placement and escalation of respiratory support. Because of the voluntary sampling strategy, unequal country representation, and differences in respondent characteristics, these findings should not be interpreted as representative estimates of national or global practice. Further multicenter observational studies using more representative sampling strategies are needed to determine whether the observed practice differences are associated with differences in patient outcomes.

## Figures and Tables

**Table 1:** Respondent and institutional characteristics according to respondent group.

Characteristic	International Respondents (n = 284)	Italian Respondents (n = 225)
Age group		
≤30 years	57 (20.1%)	31 (13.8%)
31–40 years	120 (42.3%)	98 (43.6%)
41–50 years	66 (23.2%)	63 (28.0%)
51–60 years	37 (13.0%)	21 (9.3%)
61–69 years	4 (1.4%)	12 (5.3%)
Gender		
Female	92 (32.4%)	84 (37.3%)
Male	186 (65.5%)	138 (61.3%)
Other/prefer not to answer	6 (2.1%)	3 (1.3%)
Years since specialization		
Not yet specialized	30 (10.6%)	71 (31.6%)
<5 years	79 (27.8%)	46 (20.4%)
5–10 years	66 (23.2%)	28 (12.4%)
11–15 years	48 (16.9%)	34 (15.1%)
16–20 years	23 (8.1%)	20 (8.9%)
>20 years	38 (13.4%)	26 (11.6%)
Area of specialization		
Acute care surgery	3 (1.1%)	13 (5.8%)
General surgery	12 (4.2%)	8 (3.6%)
Thoracic surgery	30 (10.6%)	25 (11.1%)
Anesthesiology	33 (11.6%)	71 (31.6%)
Intensive care	59 (20.8%)	19 (8.4%)
Internal medicine	10 (3.5%)	12 (5.3%)
Pulmonology	117 (41.2%)	49 (21.8%)
Emergency medicine	9 (3.2%)	15 (6.7%)
Traumatology	9 (3.2%)	2 (0.9%)
Pediatrics	2 (0.7%)	11 (4.9%)
Type of hospital		
First-level hospital	24 (8.4%)	33 (14.7%)
Second-level hospital	39 (13.7%)	66 (29.3%)
Third-level hospital	215 (75.7%)	119 (52.9%)
Other	6 (2.1%)	7 (3.1%)
Main working setting		
Department of medicine	11 (3.9%)	7 (3.1%)
Pulmonology department	109 (38.4%)	42 (18.7%)
Emergency medicine	12 (4.2%)	23 (10.2%)
Intensive care unit	81 (28.5%)	77 (34.2%)
Semi-intensive care unit	3 (1.1%)	4 (1.8%)
Other medical department	13 (4.6%)	10 (4.4%)
Pediatric department	2 (0.7%)	8 (3.6%)
Emergency surgery unit	13 (4.6%)	27 (12.0%)
Other surgical department	40 (14.1%)	27 (12.0%)
Self-reported expertise in blunt chest trauma		
Very expert	44 (15.5%)	15 (6.7%)
Quite expert	137 (48.2%)	64 (28.4%)
Non-expert	103 (36.3%)	146 (64.9%)
Hospital size		
50–200 beds	79 (27.8%)	58 (25.8%)
201–800 beds	105 (37.0%)	113 (50.2%)
>800 beds	100 (35.2%)	54 (24.0%)
Institutional resources		
Acute Pain Service available	170/284 (59.9%)	169/225 (75.1%)
Internal NIV protocol available	165/284 (58.1%)	65/225 (29.0%)
Internal rib fracture protocol available	127/284 (44.8%)	71/225 (31.6%)

Values are presented as n (%). NIV, non-invasive ventilation.

**Table 2:** Diagnostic and initial management strategies in blunt chest trauma.

Characteristic	International Respondents (n = 284)	Italian Respondents (n = 225)
Initial imaging modality in hemodynamically stable patients		
Chest X-ray	151 (53.2%)	75 (33.5%)
CT scan	109 (38.4%)	105 (46.9%)
Ultrasound	24 (8.5%)	44 (19.6%)
Initial imaging modality in hemodynamically unstable patients		
Chest X-ray	67 (23.6%)	39 (17.3%)
CT scan	69 (24.3%)	97 (43.1%)
Ultrasound	148 (52.1%)	89 (39.6%)
Management of pneumothorax in stable patients		
Observation only	60 (21.2%)	61 (27.4%)
Needle aspiration	34 (12.0%)	11 (4.9%)
Chest tube placement	186 (65.7%)	148 (66.4%)
VATS	4 (1.1%)	5 (1.4%)
Management of pneumothorax in unstable patients		
Emergency department thoracotomy	56 (19.8%)	44 (19.6%)
Chest tube placement	200 (70.7%)	159 (71.0%)
VATS	28 (9.5%)	22 (9.4%)

Values are presented as n (%). CT, computed tomography; VATS, video-assisted thoracoscopic surgery.

**Table 3:** Analgesic strategies, respiratory support, and rib fracture management in blunt chest trauma.

Characteristic	International Respondents (n = 284)	Italian Respondents (n = 225)
Preferred analgesic strategy		
Intravenous opioids	97 (34.2%)	57 (25.5%)
Regional anesthesia techniques	97 (34.2%)	105 (46.9%)
Intravenous non-opioid analgesics	82 (28.9%)	60 (26.8%)
Other	8 (2.8%)	2 (0.9%)
missing	0	1
Initial ventilatory support strategy		
Non-invasive ventilation (NIV)	136 (47.9%)	84 (37.7%)
Invasive mechanical ventilation (IMV)	40 (14.1%)	13 (5.8%)
CPAP (full-face mask)	27 (9.5%)	20 (9.0%)
CPAP (helmet)	1 (0.4%)	15 (6.7%)
High-flow nasal oxygen (HFNO)	78 (27.5%)	89 (39.9%)
Other	2 (0.7%)	2 (0.9%)
missing	0	2
Preferred NIV mode		
CPAP	99/241 (41.1%)	124/210 (59.0%)
BiPAP	134/241 (55.6%)	86/210 (41.0%)
Other	8/241(3.3%)	0 (0.0%)
missing	43	15
Indications for transition from NIV to IMV *		
Worsening respiratory distress	159/239 (66.5%)	126/211 (59.7%)
Hemodynamic instability	120/239 (50.2%)	88/211 (41.7%)
Persistent hypoxemia	115/239 (48.1%)	87/211 (41.2%)
No improvement with NIV	144/239 (60.3%)	116/211 (55.0%)
Need for surgical intervention	66 /239(27.6%)	37/211 (17.5%)
Other	4/239 (1.7%)	4 /211(1.90%)
missing	45	14
Pneumothorax as an absolute contraindication to NIV		
Yes	85 (37.78%)	50/208(24.04%)
No	140 (62.22%)	158/208 (75.96%)
missing	0	17
Use of scores for ICU admission		
Yes	109/227 (48.02%)	101/208 (48.56%)
No	118/227 (51.98%)	107/208 (51.44%)
missing	57	17
Specialist responsible for rib fracture management		
Thoracic surgeon	135/224 (60.27%)	123 /203(60.59%)
Traumatologist	26/224 (11.61%)	39/203 (19.21%)
General surgeon	63/224 (28.13%)	41/203 (20.20%)
missing	60	22
Main factor guiding rib fracture treatment		
Flail chest	166/222 (74.77%)	14/199 (7.04%)
Age	3/222 (1.35%)	142/199 (71.36%)
Pain with inadequate control	22/222 (9.91%)	21/199 (10.55%)
Chest deformity	10/222 (4.50%)	14/199 (7.04%)
Compound fracture of >1 rib	21/222 (9.46%)	8/199 (4.02%)
missing	62	26

Values are presented as n (%). BiPAP, bilevel positive airway pressure; CPAP, continuous positive airway pressure; HFNO, high-flow nasal oxygen; ICU, intensive care unit; IMV, invasive mechanical ventilation; NIV, non-invasive ventilation. * multiple answers were allowed.

## Data Availability

The data supporting the findings of this study are available from the corresponding author upon reasonable request.

## Supplementary Materials

The following supporting information can be downloaded at: https://www.mdpi.com/article/10.3390/jcm15197668/s1, Supplementary Material S1: Chest Trauma Study Group International Survey; Supplementary Material S2: Chest Trauma Study Group Collaborators.

## References

[1] Battle C., O’Neill C., Newey L., Barnett J., O’Neill M., Hutchings H. (2021). A survey of current practice in UK emergency department management of patients with blunt chest wall trauma not requiring admission to hospital. Injury.

[2] Liman S.T., Kuzucu A., Tastepe A.I., Ulasan G.N., Topcu S. (2003). Chest injury due to blunt trauma. Eur. J. Cardiothorac. Surg..

[3] Battle C.E., Hutchings H., Evans P.A. (2012). Risk factors that predict mortality in patients with blunt chest wall trauma: A systematic review and meta-analysis. Injury.

[4] Lundin A., Akram S.K., Berg L., Göransson K.E., Enocson A. (2022). Thoracic injuries in trauma patients: Epidemiology and its influence on mortality. Scand. J. Trauma Resusc. Emerg. Med..

[5] Pieracci F.M., Leasia K., Bauman Z., Eriksson E.A., Lottenberg L., Majercik S., Powell L., Sarani B., Semon G., Thomas B. (2020). A multicenter, prospective, controlled clinical trial of surgical stabilization of rib fractures in patients with severe, nonflail fracture patterns (Chest Wall Injury Society NONFLAIL). J. Trauma Acute Care Surg..

[6] Sawyer E., Wullschleger M., Muller N., Muller M. (2022). Surgical rib fixation of multiple rib fractures and flail chest: A systematic review and meta-analysis. J. Surg. Res..

[7] Kourouche S., Buckley T., Munroe B., Curtis K. (2018). Development of a blunt chest injury care bundle: An integrative review. Injury.

[8] Curtis K., Kourouche S., Asha S., Considine J., Fry M., Middleton S., Mitchell R., Munroe B., Shaban R.Z., D’Amato A. (2021). Impact of a care bundle for patients with blunt chest injury (ChIP): A multicentre controlled implementation evaluation. PLoS ONE.

[9] Marasco S., Lee G., Summerhayes R., Fitzgerald M., Bailey M. (2015). Quality of life after major trauma with multiple rib fractures. Injury.

[10] Baker E., Battle C., Banjeri A., Carlton E., Dixon C., Ferry J., Hopkins P., Jones R., Murrells T., Norton C. (2021). Prospective observational study to examine health-related quality of life and develop models to predict long-term patient-reported outcomes 6 months after hospital discharge with blunt thoracic injuries. BMJ Open.

[11] Gooseman M.R., Cowen M.E., Tentzeris V., Qadri S.S.A., Brunelli A., Pompili C. (2023). Patient reported outcomes in thoracic trauma—Lesson learned from thoracic oncology. J. Thorac. Dis..

[12] Coccolini F., Cremonini C., Moore E.E., Civil I., Balogh Z., Leppaniemi A., Horer T., Reva V., Ball C., Kirkpatrick A.W. (2025). Thoracic trauma WSES-AAST guidelines. World J. Emerg. Surg..

[13] Sermonesi G., Bertelli R., Pieracci F.M., Balogh Z.J., Coimbra R., Galante J.M., Hecker A., Weber D., Bauman Z.M., Kartiko S. (2024). Surgical stabilization of rib fractures (SSRF): The WSES and CWIS position paper. World J. Emerg. Surg..

[14] Yarad C.A., Lewis D., Hardy B.M., King K.L., Fischer A., Balogh Z.J. (2025). Time to computed tomography for major trauma patients: 10-year trends at a level-1 trauma centre. ANZ J. Surg..

[15] Bresgen T.U., Salinaro F., Barcella B., Perlini S., Mascherona I., Di Pietro S. (2025). Acute pain management of rib fractures: A narrative review. Injury.

[16] Kim M., Moore J.E. (2020). Chest trauma: Current recommendations for rib fractures, pneumothorax, and other injuries. Curr. Anesthesiol. Rep..

[17] Peek J., Beks R.B., Kingma B.F., Marsman M., Ruurda J.P., Houwert R.M., Leenen L.P.H., Hietbrink F., de Jong M.B. (2019). Epidural analgesia for severe chest trauma: An analysis of current practice on the efficacy and safety. Crit. Care Res. Pract..

[18] Sousa G., Barbosa Ribeiro A., Segura-Grau E., Santos C. (2024). Improving thoracic trauma care: Locoregional analgesia in the intensive care unit. Cureus.

[19] Gadsden J., Warlick A. (2015). Regional anesthesia for the trauma patient: Improving patient outcomes. Local Reg. Anesth..

[20] Lovisari F., Favarato M., Giovannini I., Giudici R., Fumagalli R. (2020). Chest wall pain management after chest wall trauma. J. Vis. Surg..

[21] Peek J., Smeeing D.P.J., Hietbrink F., Houwert R.M., Marsman M., de Jong M.B. (2019). Comparison of analgesic interventions for traumatic rib fractures: A systematic review and meta-analysis. Eur. J. Trauma Emerg. Surg..

[22] Chiumello D., Coppola S., Froio S., Gregoretti C., Consonni D. (2013). Noninvasive ventilation in chest trauma: Systematic review and meta-analysis. Intensive Care Med..

[23] Werle R.W., Delevati A.L., Righi N.C., Nunes G.S., Signori L.U., da Silva A.M.V. (2026). Noninvasive ventilation in adults with blunt chest trauma: A meta-analysis of randomized clinical trials. Braz. J. Phys. Ther..

[24] Dufraigne A., Bonjean P., Gergele L., Morel J., Thiery G., Beuret P. (2025). Noninvasive ventilation in chest trauma-related acute respiratory failure related to chest trauma: Efficacy and risk of pneumothorax. Eur. J. Trauma Emerg. Surg..

[25] Duggal A., Perez P., Golan E., Tremblay L., Sinuff T. (2013). Safety and efficacy of noninvasive ventilation in patients with blunt chest trauma: A systematic review. Crit. Care.

[26] Battle C., Pelo C., Hsu J., Driscoll T., Whitbeck S., White T., Webb M. (2023). Expert consensus guidance on respiratory physiotherapy and rehabilitation of patients with rib fractures: An international, multidisciplinary e-Delphi study. J. Trauma Acute Care Surg..

[27] Van Diepen M.R., Van Lieshout E.M.M., Van Bennekom A.W., Verhofstad M.H.J., Knops S.P., Vermeulen J., Wijffels M.M.E. (2025). The value of bronchodilators in patients with posttraumatic rib fractures (RIB-Inhalation): A multicenter prospective cohort study. Surgery.

[28] Chau H.S., Gutkin P.M., Knight A.W., Forrester J.D. (2025). Contemporary management of pulmonary contusions: A narrative review. Curr. Chall. Thorac. Surg..

