# Supplementary material for: Heterogeneity in Self-Reported Management of Blunt Chest Trauma: An International Cross-Sectional Survey
Source: J Clin Med. 2026 Oct 3;15(19):7668. doi: 10.3390/jcm15197668 (PMC13666495; doi:10.3390/jcm15197668)
Supplement: Supplementary file 1 [file jcm-15-07668-s001.zip › Supplementary_Material_S1.pdf]

Supplementary Material S1
Chest Trauma Study Group International Survey

1. What is your age group?
- ≤30
- 31-40
- 41-50
- 51-60
- 61-69
- ≥70

2. Which gender identity do you most identify with?
- Female
- Male
- Gender variant/non conforming
- Prefer not to answer

3. How long have you been specialized?
- Not yet specialized
- <5 years
- 5-10 years
- 11-15 years
- 16-20 years
- >20 years

4. What is your area of specialization?
- Acute care surgeon
- General surgeon
- Thoracic surgeon
- Anestesiologist
- Intensive care phisician
- Internal medicine
- Pneumology
- Emergency phisician
- Traumatology
- Pediatrician

5. In which country do you work?
- Country (drop-down list)

6. In which type of hospital do you work?
- First level hospital (Few specialties—mainly internal medicine, obstetrics and gynecology, pediatrics, and general surgery)
- Second level Hospital (More differentiated by function, with as many as 5 to 10 clinical specialties)
- Third level Hospital (Highly specialized staff and technical equipment)
- Other (to be specified)

7. In which main setting do you work?
- Department of medicine
- Pneumology department
- Department of Emergency Medicine
- Intensive care unit
- Semi intensive care unit
- Other medical department
- Pediatric department

- Emergency surgery unit
- Other surgical department

8. Do you consider yourself an expert in the management of chest blunt thoracic trauma?

- Yes I consider myself very expert
- Yes I consider myself quite expert
- No I do not consider myself an expert

9. How many beds are in your hospital?

- 50-200
- 201-800
- >800

10. In your clinical practice, what is the initial imaging modality of choice for hemodinamically stable patients with blunt thoracic trauma?

- Chest X-ray
- CT scan
- Ultrasound

11. In your clinical practice, what is the initial imaging modality of choice for hemodinamically unstable patients after blunt thoracic trauma?

- Chest X-ray
- CT scan
- Ultrasound

12. How do you manage patients with a stable hemodynamic status and evidence of pneumothorax on imaging (>2 cm) after blunt thoracic trauma?

- Observation only
- Needle aspiration
- Chest tube placement
- Video-assisted thoracoscopic surgery (VATS)

13. What is your preferred approach for the management of patients with a hemodynamically unstable blunt thoracic trauma?

- Emergency department thoracotomy
- Chest tube
- Video-assisted thoracoscopic surgery (VATS)

14. Do you have an acute pain service available in you hospital?

- Yes
- No

15. In hemodinamic stable patients with multiple rib fractures and respiratory compromise, what is your preferred method of pain control?

- Intravenous opioids
- Regional anesthesia techniques (e.g. epidural or intercostal nerve blocks)
- Intravenous non-opioid analgesics
- Other (to be specified)

16. In your clinical practice, what is the most commonly used device for the initial management of intermediate blunt chest trauma requiring respiratory support?

- Non-invasive ventilation (NIV)
- Invasive mechanical ventilation (IMV)
- Continuous positive airway pressure (CPAP) (full face mask)
- Continuous positive airway pressure (CPAP) (helmet)
- High-Flow Nasal Oxigen (HFNO)
- Other (to be specified)

17. Do you have an internal protocol to guide you to use NIV in hemodynamic stable patients?

- Yes
- No

18. If you have an internal protocol, what are the main factors taken into consideration?

- Arterial blood gas analysis
- Oxygen saturation levels
- Respiratory rate and effort
- Chest X-ray or CT scan findings
- Score
- All of the above

19. Do you use a score to determine if a patient is a candidate for one type of ventilator support?

- Yes
- No

20. If yes, which one do you use?

- Chest trauma score
- Abbreviated Injury Scale
- Pulmonary contusion score
- Wagner Score
- Thoracic trauma severity score

21. What is your preferred mode of NIV for patients with blunt thoracic trauma?

- Continuous positive airway pressure (CPAP)
- Bilevel positive airway pressure (BiPAP)
- Other (to be specified)

22. Do you use any specific ventilator settings or parameters for patients with blunt thoracic trauma on NIV?

- Yes
- No

23. In your experience, what are the most common indications for a patient with blunt chest trauma to transition from NIV to IMV?

- Worsening respiratory distress
- Hemodynamic instability
- Persistent hypoxemia
- Failure to improve with NIV
- Need of surgical intervention
- Other (to be specified)

24. Do you use routinely any form of respiratory assistance in hemodinamically stable patients to prevent morbidity from lung contusion (pneumonia, pleural effusion)?

- Yes
- No

  If yes, please specify:

  - Non-invasive ventilation (NIV)
  - Continuous positive airway pressure (CPAP) (helmet)
  - Continuous positive airway pressure (CPAP) (full mask)
  - High-Flow Nasal Oxigen (HFNO)
  - Other (to be specified)

25. In your clinical practice, do you consider PNX an absolute contraindication to NIV?

- Yes
- No

26. What adjunctive therapies do you routinely use in patients with blunt thoracic trauma on NIV?

- None of the above therapies
- Physiotherapy

- Bronchodilators
- Diuretics

27. Do you use specific clinical or radiological criteria to determine which patients with blunt force trauma require admission to the ICU?

- Yes
- No

28. Do you use one of the following scores to determine whether a patient needs an intensive care unit admission (Chest trauma score, Abbreviated Injury Scale, Pulmonary contusion score, Wagner Score, Thoracic trauma severity score)?

- Yes
- No

29. If yes, which one do you use?

- Chest trauma score
- Abbreviated Injury Scale
- Pulmonary contusion score
- Wagner Score
- Thoracic trauma severity score

30. In case of rib fractures do you follow an internal diagnostic/treatment flow-chart to treat rib fractures?

- Yes
- No

31. Who treats rib fractures at your institution?

- Thoracic surgeons
- Traumatologist
- General surgeon

32. What is the most important factor in deciding to treat compound rib fractures?

- Flail chest
- Age
- Pain with not adequate control
- Chest deformity
- Compound fracture of >1 rib
