# Supplementary material for: Heterogeneity in Self-Reported Management of Blunt Chest Trauma: An International Cross-Sectional Survey
Source: J Clin Med. 2026 Oct 3;15(19):7668. doi: 10.3390/jcm15197668 (PMC13666495; doi:10.3390/jcm15197668)
Supplement: Supplementary file 1 [file jcm-15-07668-s001.zip › Supplementary_Material_S2.pdf]

Supplementary Material S2

Chest Trauma Study Group Collaborators

The following individuals are members of the Chest Trauma Study Group who contributed to this multicenter survey.

1. **Davide Piredda** — Anesthesia and General Intensive Care Unit, AOU Sassari, Sassari, Italy
2. **Luca Schiaffino** — Anesthesia and General Intensive Care Unit, AOU Sassari, Sassari, Italy
3. **Giuseppe Lacerra** — Interventional Pulmonology Unit, AORN Ospedali dei Colli – Monaldi Hospital, Naples, Italy
4. **Anna Annunziata** — Respiratory Pathophysiology Unit, AORN Ospedali dei Colli – Monaldi Hospital, Naples, Italy
5. **Marcello Sorrentino** — Anesthesia and Intensive Care, Fatebenefratelli Hospital, Naples, Italy
6. **Paolo Smaldone** — Department of Anesthesia and Intensive Care, University of Campania “Luigi Vanvitelli”, Naples, Italy
7. **Salvatore Romano** — Department of Anesthesia and Intensive Care, University of Campania “Luigi Vanvitelli”, Naples, Italy
8. **Carlo Iadevaia** — Department of Pneumology, University of Campania “Luigi Vanvitelli”, Naples, Italy
9. **Francesco Cerqua** — Department of Pneumology, University of Campania “Luigi Vanvitelli”, Naples, Italy
10. **Giuseppe Pelella** — Department of Congenital Cardiac Surgery, Leeds Teaching Hospitals NHS Trust, Leeds, United Kingdom
11. **Lucio Maresca** — Department of Surgery, AORN Ospedali dei Colli – Monaldi Hospital, Naples, Italy
12. **Safy Kaddah** — Department of Pulmonology, Kasr Al Ainy Hospital, Cairo University, Cairo, Egypt
13. **Paolo Onida** — Department of Anesthesia, Azienda Ospedaliera Universitaria di Cagliari, Cagliari, Italy
14. **Nicolò Pecorelli** — Division of Pancreatic Surgery, IRCCS San Raffaele Scientific Institute, Milan, Italy
15. **Giovanna Esposito** — Department of Anesthesia and Intensive Care, CTO Hospital, Naples, Italy
16. **Michela Riccio** — Anesthesia and Hyperbaric Medicine Center, AORN Santobono-Pausilipon, Naples, Italy
17. **Emilio Di Costanzo** — Department of Anesthesia and Post-Operative Intensive Care Unit, AORN Ospedali dei Colli – Monaldi Hospital, Naples, Italy
18. **Luca Crisanti** — Sapienza University of Rome, Sant’Andrea Hospital, Postgraduate School of Emergency Medicine, Department of Emergency Medicine, Rome, Italy
19. **Gabriele Valli** — UOC Pronto Soccorso e Medicina d’Urgenza, Ospedale di Anzio, ASL Roma 6, Rome, Italy
20. **Elisa Cennamo** — UOC Pronto Soccorso e Medicina d’Urgenza, A.O. San Giovanni Addolorata, Rome, Italy
21. **Francesca De Marco** — UOC Pronto Soccorso e Medicina d’Urgenza, Ospedale San Filippo Neri, Rome, Italy
22. **Dino Casazza** — Department of Thoracic Surgery, AORN Ospedali dei Colli – Monaldi Hospital, Naples, Italy
23. **Filomena Napolitano** — Department of Thoracic Surgery, AORN Ospedali dei Colli – Monaldi Hospital, Naples, Italy
24. **Ernesto Serpe** — Department of Pulmonology, Kore University of Enna, Enna, Italy
25. **Rakhee Sodhi Khanduri** — Department of Respiratory Medicine, Swami Rama Himalayan University, Dehradun, Uttarakhand, India
26. **Abhilasha Motghare** — Department of Anesthesiology, All India Institute of Medical Sciences (AIIMS) Rajkot, Rajkot, Gujarat, India
27. **Pulkit Gupta** — Department of Pulmonary Medicine, Dr. Ram Manohar Lohia Institute of Medical Sciences, Lucknow, Uttar Pradesh, India
28. **Tanmoy Ghatak** — Department of Emergency Medicine, Sanjay Gandhi Postgraduate Institute of Medical Sciences, Lucknow, Uttar Pradesh, India
29. **Antriksh Srivastava** — Department of Respiratory Medicine, TS Misra Medical College and Hospital, Lucknow, Uttar Pradesh, India
30. **Madhurmay** — Department of Respiratory Medicine, Maharani Laxmi Bai Medical College, Jhansi, Uttar Pradesh, India
31. **Nitin Rai** — Department of Critical Care Medicine, Healthcity Vistaar Superspeciality Hospital, Lucknow, Uttar Pradesh, India

32. **Jyoti Narayan Sahoo** — Department of Critical Care Medicine, Apollo Hospitals, Bhubaneswar, Odisha, India
33. **Deepak Dwivedi** — Department of Surgery, Military Hospital Ahmednagar, Maharashtra, India
34. **Kunal Deokar** — Department of Pulmonary Medicine, All India Institute of Medical Sciences (AIIMS) Rajkot, Rajkot, Gujarat, India
35. **Arun Sharma** — Department of Critical Care Medicine, Rungta Hospital, Jaipur, Rajasthan, India
36. **Kiran Vishnu Narayan** — Department of Pulmonary Medicine, Government Medical College, Kollam, Kerala, India
37. **Purujit Choudhury** — Department of Surgery, Gauhati Medical College and Hospital, Guwahati, Assam, India
38. **Adele Molinaro** — Intensive Care Unit for Trauma and Extracorporeal Support, Careggi University Hospital, Florence, Italy
39. **Jianpeng Zhang** — DAMO Academy, Alibaba Group, Hangzhou, China; Hupan Laboratory, Hangzhou, China; College of Computer Science and Technology, Zhejiang University, Hangzhou, China
40. **Gennaro Martucci** — Department of Anesthesia and Intensive Care, Istituto Mediterraneo per i Trapianti e Terapie ad alta Specializzazione (IRCCS-ISMETT), Palermo, Italy
41. **Clelia Esposito** — Department of Anesthesia and Post-Operative Intensive Care Unit, AORN Ospedali dei Colli – Monaldi Hospital, Naples, Italy
42. **Gianmaria Chicone** — Department of Anesthesia and Intensive Care, Ospedale del Mare, Naples, Italy
43. **Antonio Tommasello** — Emergency and Acceptance Department, Anesthesia, Emergency and Burn Intensive Care Unit, A.O.R.N. Cardarelli, Naples, Italy
44. **Roberto Andini** — Internal Medicine, University of Campania Luigi Vanvitelli; AORN Ospedali dei Colli – Monaldi Hospital, Naples, Italy
45. **Marianna Tamborrino** — Department of Anesthesia and Intensive Care, CTO Hospital, Naples, Italy
46. **Eldhos Jacob** — Department of Respiratory Medicine, Father Muller Medical College Hospital, Mangaluru, Karnataka, India
47. **Francesca Simioli** — Respiratory Pathophysiology Unit, AORN Ospedali dei Colli – Monaldi Hospital, Naples, Italy; Federico II University, Naples, Italy
48. **Antonietta Coppola** — Respiratory Pathophysiology Unit, AORN Ospedali dei Colli – Monaldi Hospital, Naples, Italy; Federico II University, Naples, Italy
49. **Berkan Basançelebi** — Department of Electroneurophysiology, Vocational School, İstanbul Medipol University, İstanbul, Türkiye
50. R**oberto Giurazza-**Department of Woman, Child, General and Specialized Surgery, University of Campania "Luigi Vanvitelli", Naples, Italy,Department of Critical Care, AORN Ospedali dei Colli, Naples, Italy
